# Identification of a metabolic–immune crosstalk in Still’s disease: monocyte/macrophage-derived immunometabolite itaconate dictates hepatic immunopathology via the CXCL10–CD8 T cell axis

**DOI:** 10.1038/s12276-026-01751-x

**Published:** 2026-06-05

**Authors:** Junna Ye, Fan Wang, Zhuochao Zhou, Jinchao Jia, Ziang Wu, Yijun You, Nadiem Atiq, Yutong Su, Huihui Chi, Jianfen Meng, Mengyan Wang, Yuning Ma, Shirin Hosseini, Martin Korte, Jialin Teng, Chengde Yang, Karsten Hiller, Qiongyi Hu, Wei He, Yue Sun

**Affiliations:** 1 Department of Rheumatology and Immunology, Ruijin Hospital, Shanghai Jiao Tong University School of Medicine, Shanghai, China; 2 Department of Critical Care Medicine, Ruijin Hospital, Shanghai Jiao Tong University School of Medicine, Shanghai, China; 3 Braunschweig Integrated Centre of Systems Biology (BRICS), Technische Universität Braunschweig, Braunschweig, Germany; 4 Zoological Institute, Technische Universität Braunschweig, Braunschweig, Germany

**Keywords:** Chronic inflammation, Inflammatory diseases

## Abstract

Still’s disease (SD) is a chronic and systemic autoinflammatory disorder, with the possibility of resulting in life-threatening complications, including macrophage activation syndrome (MAS). The metabolic–immune interplay underlying the immunopathology of SD/MAS remains largely unexplored. In this study, we identified itaconate — a myeloid cell-specific metabolite derived from the tricarboxylic acid cycle via the enzyme ACOD1 — as a dual regulator of inflammation and chemokine-driven tissue injury in SD/MAS. Clinical metabolomics revealed elevated serum itaconate in patients with SD, attributable to peripheral blood monocytes and correlated with disease severity. This was consolidated by the identification of the *Acod1*–itaconate axis in monocytes and macrophages in both a mouse model of MAS and in vitro cell cultures. Although itaconate suppressed IL-1β, IL-6, CXCL1 and CCL2 in vitro, it paradoxically amplified CXCL10 secretion in vitro and in vivo. This was in line with the observations of elevated plasma CXCL10 levels in patients with MAS. In the CpG ODN 1826-induced MAS mouse model, ablation of *Acod1* ameliorated disease manifestations and hepatic inflammation, accompanied by a reduced CXCL10 level as well as attenuated hepatic infiltration of CD8^+^ T cells. Collectively, our study reveals a previously unrecognized metabolic–immune crosstalk in AOSD/MAS, positioning monocyte/macrophage-derived itaconate as a dual regulator that suppresses canonical pro-inflammatory cytokines while licensing CXCL10-mediated CD8^+^ T cell-driven tissue injury. Therefore, discovery from this study calls for scrutiny of an itaconate-based anti-inflammatory strategy in chronic inflammatory diseases.

## Itaconate Reveals Immune Metabolism in Still’s Disease

Still’s disease (SD) is a systemic autoinflammatory disorder marked by severe inflammation and complications such as macrophage activation syndrome (MAS). Current treatments often prove insufficient, necessitating new therapeutic targets. This study explores the role of itaconate, a metabolite produced by monocytes and macrophages, in SD/MAS. Researchers found elevated itaconate levels in patients with SD, correlating with disease severity. In a mouse model, itaconate enhanced CXCL10 production, a chemokine linked to CD8^+^ T cell recruitment, while suppressing other inflammatory cytokines. Genetic ablation of itaconate production reduced MAS severity and inflammation, highlighting the itaconate–CXCL10 axis as a potential therapeutic target. These findings suggest that targeting the itaconate pathway could modulate immune responses in SD/MAS, offering a novel approach to treatment. Future research should explore the precise mechanisms of the complex effects of itaconate and its potential in other inflammatory conditions.

This summary was initially drafted using artificial intelligence, then revised and fact-checked by the author.

## Introduction

Still’s disease (SD) is a systemic autoinflammatory disorder characterized by cytokine storm-mediated complications, including macrophage activation syndrome (MAS) — a life-threatening hyperinflammatory state that often proves refractory to IL-1β/IL-6-targeted therapies^1–3^. The limited efficacy of current therapies in severe SD/MAS underlines the necessity to identify novel drivers of immunopathology. Emerging evidence implicates aberrant sensing of pathogen- and damage-associated molecular patterns via Toll-like receptors (TLRs) in initiating SD flares^4,5^. This triggers NLRP3 inflammasome activation and excessive production of IL-1β, IL-6 and IL-18, while simultaneously activating both type I (IFN-α/β) and type II (IFN-γ) interferon pathways^1,6^. These responses synergistically drive disease progression by amplifying inflammatory cascades. Notably, viral infections such as echovirus 7 (activating TLR3), parvovirus B19 and cytomegalovirus (both engaging TLR9) have been implicated in triggering SD through innate immune dysregulation^5,7,8^. However, the precise molecular mechanisms linking these innate immune triggers to the dysregulated adaptive immune responses remain incompletely understood.

Innate and adaptive immune dysregulation in SD manifests as expanded classical and non-classical monocyte subsets, heightened neutrophil activation and skewed lymphocyte populations — including elevated effector CD4^+^ (notably Th17) and CD8^+^ T cells alongside reduced B and NK/NKT cells^9,10^. Chemokine networks critically orchestrate these immune cell shifts in SD: elevated levels of monocyte-derived CCL2 and CCL3 drive further myeloid recruitment via CCR2/CCR5, whereas neutrophil-associated CXCL8 (IL-8), which is consistently upregulated in SD, amplifies tissue neutrophilia^11,12^. CX3CL1 and CXCL13, both markedly elevated in SD sera, contribute to disease-specific immune trafficking — CX3CL1 recruits monocytes/neutrophils via CX3CR1, and CXCL13 directs CXCR5^+^ B cell migration^11,13^. In particular, CXCL10 — an IFN-inducible chemokine with pronounced elevation in SD — recruits CXCR3^+^ T cells and exhibits the strongest correlation with disease severity, positioning it as a central mediator bridging innate triggers (e.g. TLR activation) to adaptive immune infiltration^13–15^. Collectively, these chemokine–immune cell axes constitute a self-amplifying inflammatory loop that exacerbates tissue damage, resulting in disease severity in SD. A critical, unresolved question is what drives the aberrant production of these pathogenic chemokines in SD/MAS.

Studies in the fast-rising field of immunometabolism have revealed various metabolites as novel regulators of immune-cell functions. These metabolites (recently termed immunometabolites) are demonstrated to impact immune cells beyond the traditional role of metabolism in bioenergetics and biosynthesis, instead, via regulations on gene expression, signalling pathways, epigenetics, etc.^16,17^. Concomitantly, immune cells reprogramme their cellular metabolism to accumulate and/or secrete these immunometabolites, enabling their respective regulatory actions on an intracellular or intercellular basis^18,19^. Among these immunometabolites, itaconate is presumably the most noticeable one during the last decade. Being the most accumulated metabolite in the lipopolysaccharide (LPS)-stimulated macrophages, itaconate synthesis is derived from the tricarboxylic acid (TCA) cycle by the enzyme aconitate decarboxylase 1 (ACOD1), also known as immune-responsive gene 1 (IRG1)^18^. Multiple studies have demonstrated the anti-inflammatory role of itaconate in macrophages by using derivatives of itaconate^20–22^. Follow-up studies by us and others revealed that the original itaconate is indeed immunomodulatory, rather than simply anti-inflammatory^23–26^. Notably, we observed a boosting effect by itaconate on CXCL10 production in LPS-stimulated macrophages, along with a decreasing effect on IL-6 and IL-12β secretion^24^. On the other hand, increased itaconate levels are implicated in various disease settings, including infection, inflammation, autoimmune, cancers and metabolic complications^27,28^. Therefore, targeting itaconate or its synthetic pathway has been proposed as a potential strategy for treating these diseases, hence calling for a comprehensive understanding of the physiological and pathological actions of itaconate.

Given that SD/MAS is characterized by innate immune activation (e.g. via TLRs) and IFN signaling — processes that itaconate is known to modulate — we hypothesized that itaconate serves as a critical immunometabolic node linking innate hyperactivation to adaptive cytotoxicity in SD/MAS. In this study, we investigated itaconate-mediated metabolic–immune crosstalk in SD/MAS. We identified monocyte- and macrophage-derived itaconate in patients with SD and CpG ODN 1826-induced MAS mice, as well as the CXCL10–CD8^+^ T cell axis in patients with SD with MAS. Cellular studies confirmed CpG-induced intracellular accumulation of itaconate in monocytes and macrophages, and further established itaconate as a positive regulator of CXCL10 expression, despite its suppressive role in IL-1β and IL-6 production. Genetic ablation of itaconate production ameliorated MAS severity and systemic inflammation, accompanied by reduced CXCL10 level and CD8^+^ T cell infiltration in livers. These findings position itaconate as a metabolic orchestrator bridging innate immune hyperactivation to adaptive cytotoxicity, offering mechanistic insights into the pathogenesis of MAS and highlighting the itaconate–CXCL10 axis as a therapeutic target for immunometabolic intervention.

## Materials and methods

### Human subjects and sample collection

**Fig. 1:**
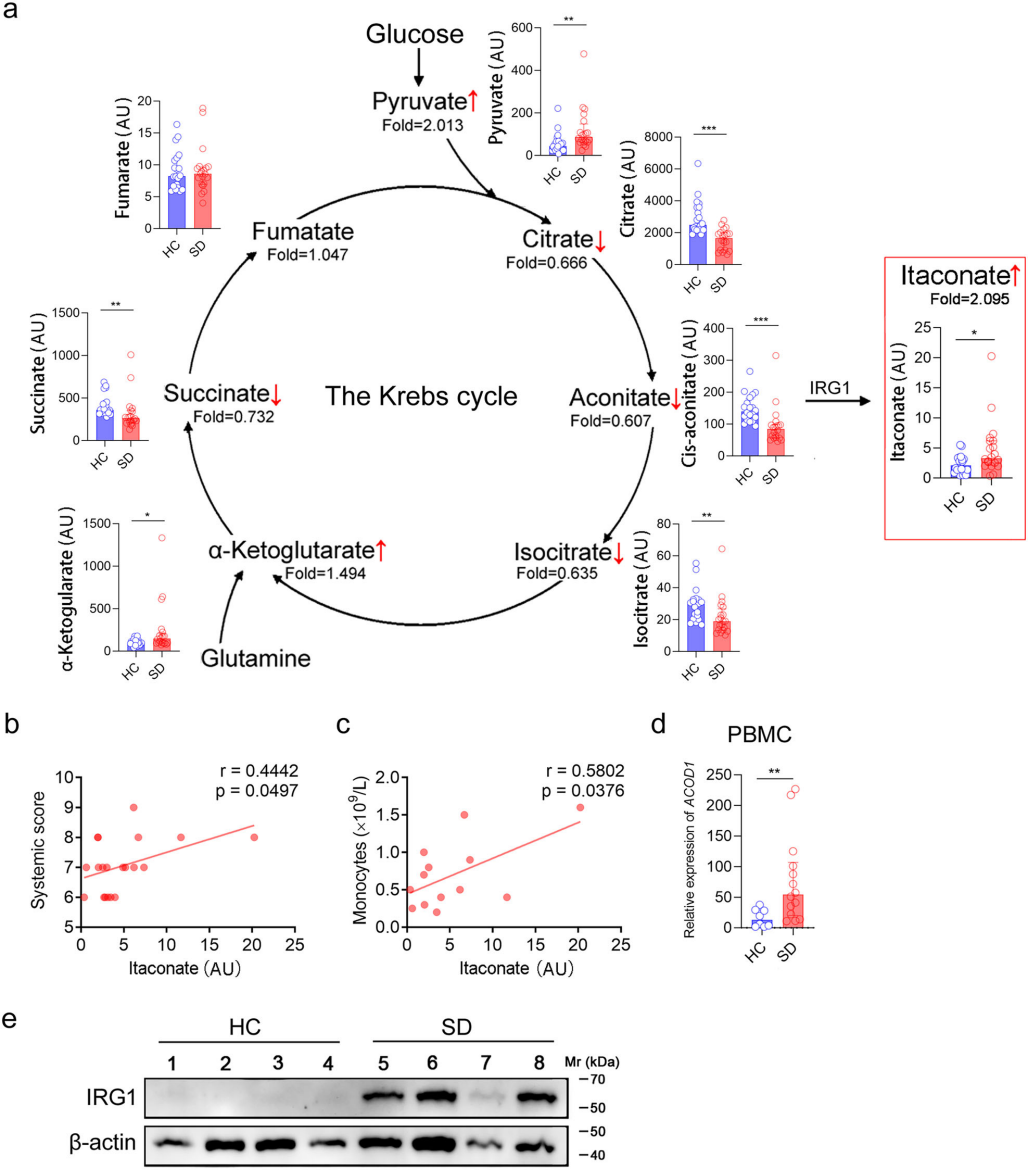
Increased levels of ACOD1/itaconate pathway in patients with active Stillʼs disease. **a** Metabolomics analysis of Krebs cycle metabolites in sera of patients with Stillʼs disease (SD) (*n* = 20) vs healthy controls (HC) (*n* = 20). **b**, **c** Correlations between serum itaconate levels and systemic score (**b**) or peripheral monocyte count (**c**) in patients with SD (*n* = 20). **d** *ACOD1* expression in peripheral blood mononuclear cells (PBMCs) from HCs (*n* = 8) and patients with SD (*n* = 14). **e**, Western blot analysis of IRG1 (53 kDa, encoded by *ACOD1*) in monocytes from HCs vs patients with SD (*n* = 4). β-actin (42 kDa) was used as an internal reference. Data (**a**) and (**d**) were presented as median with interquartile range (IQR). *P < 0.05; **P < 0.01; ***P < 0.001. AU represents arbitrary units for signal intensity.

**Fig. 2:**
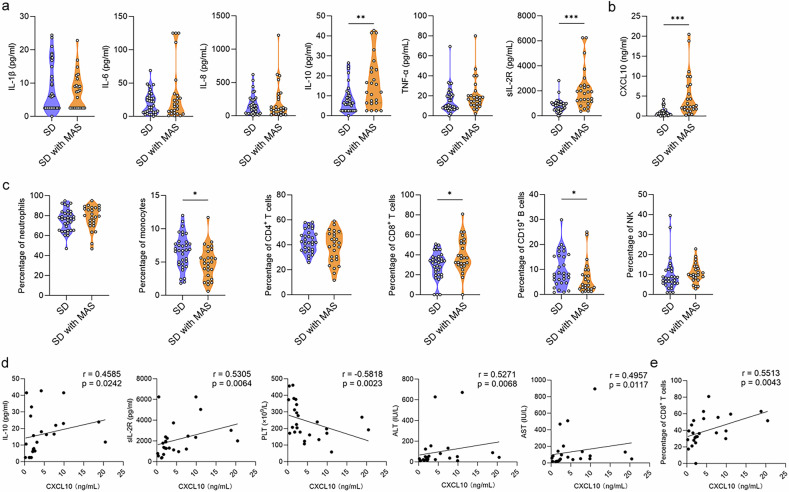
Elevated CXCL10 levels and CD8^+^ T cell percentage in peripheral blood of patients with Stillʼs disease with macrophage activation syndrome. **a** IL-1β, IL-6, IL-8, IL-10, TNF-α and sIL-2R in peripheral blood of patients with Stillʼs disease (SD) without macrophage activation syndrome (MAS) (*n* = 34) vs those with MAS (*n* = 25). **b** CXCL10 in peripheral blood of patients with SD without MAS (*n* = 34) vs those with MAS (*n* = 25). **c** Neutrophil, monocyte, CD4^+^ T, CD8^+^ T, CD19^+^ B and NK cell percentage in peripheral blood of patients with SD without MAS (*n* = 34) or those with MAS (*n* = 25). **d** Correlations between CXCL10 levels and IL-10, sIL-2R, PLT, ALT or AST in patients with SD with MAS (*n* = 25). **e** The correlation between CXCL10 levels and peripheral blood CD8^+^ T cell percentage in patients with SD with MAS (*n* = 25). Data in **a–c** are presented as violin plots (median line, kernel density estimation) with overlaid individual points. *P < 0.05; **P < 0.01; ***P < 0.001. ALT, alanine transaminase; AST, aspartate transaminase; sIL-2R, soluble IL-2 receptor.

During the period between September 2018 and March 2020, a total of 20 consecutive patients with active SD were recruited from the Department of Rheumatology and Immunology at Ruijin Hospital, Shanghai Jiao Tong University School of Medicine. The diagnosis of SD followed the Yamaguchi criteria^29^, having ruled out malignancies, infections and other autoimmune disorders. Disease severity in patients with SD was assessed using the modified Pouchot score^30^. Supplementary Table 1 details the clinical and laboratory characteristics of all participants. Prior to any therapeutic intervention, including steroids or disease-modifying anti-rheumatic drugs, sera were sampled from treatment-naïve patients with SD (without MAS) and 20 healthy control individuals (HCs) matched for age and sex. This cohort was used for serum metabolite analysis, as presented in Fig. 1. The study was approved by the Institutional Research Ethics Committee of Ruijin Hospital (protocol 2016-62) and written informed consent was secured from each participant in compliance with the Declaration of Helsinki. The samples for Fig. 2 were collected from our recently published cohort^15^.

### Isolation of peripheral blood mononuclear cells

Peripheral blood mononuclear cells (PBMCs) were isolated from EDTA-anticoagulated whole blood of patients with SD and HCs using Lymphprep™ (Axis-Shield PLC, Dundee, UK) according to the manufacturer’s protocol. Human monocytes were further enriched by 1 h adherence of PBMCs^31^.

### Transcriptomic data analysis

*ACOD1* expression in peripheral leukocyte subsets was analysed using recently reported RNA sequencing data from 18 patients with SD, 61 patients with systemic lupus erythematosus and 79 HCs (National Bioscience Database Center: E-GEAD-397)^32^. *ACOD1* expression between patients with SD with (*n* = 9) and without MAS (*n* = 3) was analysed using our recent bulk RNA-seq data (GSE247993)^15^.

### Mice

Wild-type (WT) C57BL/6 N mice (8–10 weeks old, 18–22 g; Charles River, China) and *Acod1*^−/−^ mice (Cyagen Biosciences, China) were housed under specific pathogen-free conditions. All animal procedures complied with the ARRIVE guidelines and were approved by the Ruijin Hospital Animal Care Committee (Approval No. RJ2023033). For targeted metabolomics analysis of itaconate, mesaconate and succinate, bone marrow-derived macrophages (BMDMs) were generated from wild-type C57BL/6 J mice (6–12 weeks old) bred and kept at the animal facility of the TU Braunschweig under specific pathogen-free conditions, approved by the local committees of the TU Braunschweig and the authorities (LAVES, Oldenburg, Germany §4 (09.22) TSB TU BS) according to the guidelines of the Animal Welfare Act in Germany (‘Tierschutzgesetz in der Fassung der Bekanntmachung vom 18. Mai 2006 (BGBl. I S. 1206, 1313)).

### Murine macrophage activation syndrome-like model

WT and *Acod1*^−/−^ mice (age-/weight-matched, 18-–22 g, both sexes) received intraperitoneal injections of CpG ODN 1826 (50 μg/dose in 200 μl PBS; sequence: TCCATGACGTTCCTGACGTT; phosphorothioate-modified; Sangon Biotech, China) on days 1, 3, 5, 7 and 9 (ref.^33^). Mice were euthanized by cervical dislocation on day 10. Complete blood counts (white blood cells/red blood cells/platelets) were analysed using a Sysmex pocH-100iV Diff haematology analyser (Sysmex Corporation, Japan). Sera, livers, spleens and peritoneal macrophages were collected for further studies.

### Metabolomics profiling

Monocytes/macrophages were obtained by separation of single-cell suspensions from mouse livers, spleens or peritoneal fluid with Lymphprep™ (Axis-Shield), followed by 1-h adherence to remove non-adherent cells^34^. If not mentioned otherwise, serum and intracellular itaconate levels were measured by liquid chromatography-mass spectrometry (LC-MS) using an Agilent 1290 UHPLC-6545 QTOF system (Agilent Technologies, USA). Data were processed using MassHunter software (Agilent Technologies), with peak areas normalized to internal standards. Targeted measurement of itaconate, mesaconate and succinate (Fig. 4a) were performed with gas chromatography/mass spectrometry (GC-MS) in selected ion mode as previously described^24^. Briefly, samples were derivatized with methoxylamine and N-methyl-N-(tert-butyldimethylsilyl) trifluoroacetamide, followed by GC-MS analysis with an Agilent 7890B gas chromatogram system coupled with an Agilent 5977B GC/MSD (MSD, Agilent Technologies). Data analysis was conducted utilizing Metabolite Detector software^35^.

### Flow cytometry

Hepatic leukocytes were isolated by mechanical dissociation (70-μm strainer; Beyotime, China) and enzymatic digestion (0.1 mg/ml DNase I; Solarbio, China). Cells were stained with fluorophore-conjugated antibodies: anti-CD45 (APC-Cy7, BioLegend, Inc., San Diego, CA, USA, #103116), anti-CD3 (PE-Cy7, BioLegend, #100320), anti-CD4 (FITC, BioLegend, #100406), anti-CD8 (PerCP, Invitrogen™, Thermo Fisher Scientific, Inc., Waltham, MA, USA, #46-0081-82), anti-CD11b (FITC, BioLegend, #101206), anti-F4/80 (PE, BioLegend, #123110), anti-Ly6G (APC, Invitrogen, #17-9668-82) and anti-B220 (APC, BioLegend, #103212). Data were acquired on a BD FACS Canto II (BD Biosciences, San Jose, CA, USA) and analysed using FlowJo software (version 10.7.1, FlowJo, LLC, Ashland, OR, USA). The gating strategy for flow cytometry was shown in Supplementary Fig. 1.

### Quantitative reverse transcription PCR

Total RNA was isolated from tissues/cells using TRIzol reagent (Accurate Biotechnology, China) and reverse-transcribed to cDNA using Hifair^®^ III SuperMix (Yeasen Biotechnology, China). Quantitative PCR was performed with Hieff® SYBR Green Master Mix (Yeasen Biotechnology) on a QuantStudio™ 3 Real-Time PCR system (Thermo Fisher Scientific). The primers (Supplementary Table 2) were obtained from PrimerBank (https://pga.mgh.harvard.edu/primerbank/). Gene expression was normalized to GAPDH (human) or β-actin (mouse) via 2^−ΔΔCt^.

### Western blotting

Proteins (20–40 μg) were resolved by SDS-PAGE, transferred to PVDF membranes (Millipore, Billerica, MA, USA), and probed with anti-IRG1 (1:1000; Abcam, Cambridge, UK, #ab222411), β-actin (1:1000; Servicebio, China; #GB11001) and HRP-anti-rabbit IgG (1:2000; Cell Signaling Technology, Danvers, MA, USA, #7074). Blots were visualized using a Syngene GeneGnome XRQ Chemiluminescence Imaging System (Syngene, Cambridge, UK).

### Bone marrow-derived macrophages

BMDMs were differentiated from WT/*Acod1*^−/−^ mouse bone marrow cells in RPMI-1640 (Servicebio) + 10% FBS (Servicebio) + 100 U/ml penicillin + 0.1 mg/ml streptomycin (Servicebio) + 100 ng/ml M-CSF (Novoprotein, China) for 5 days. BMDMs were stimulated with CpG ODN 1826 (1 μM; Sangon Biotech) or LPS (100 ng/ml; #L4391; Sigma-Aldrich, St. Louis, MO, USA) for the indicated time. 10 mM of itaconate (pH adjusted to 7.4; Sigma-Aldrich), 100 μM of dimethyl itaconate (DMI; Sigma-Aldrich) or 100 μM of 4-octyl itaconate (4-OI; MedChemExpress, China) were added 2 h prior to stimulation.

### Cytokine/chemokine quantification

Mouse IL-1β (#DY401), IL-6 (#DY406), IL-10 (#DY417) and CXCL10 (#DY466) levels in the supernatant from BMDMs were measured via ELISA (R&D Systems) on a BioTek Epoch Microplate Spectrophotometer (BioTek Instruments, Inc., Winooski, VT, USA). To measure IL-1β secretion from cell culture, ATP (5 mM; Topscience, China) was added 30 min prior to supernatant collection. Serum cytokines (IL-6, IL-18 and TNF-α)/chemokines (CXCL1, CXCL10 and CCL2) were analysed using LEGENDplex™ mouse macrophage/microglia panel (BioLegend, #740845) or pro-inflammatory chemokine panel 1 kits (BioLegend; #741295) on a BD FACS Canto II. The data were analysed by LEGENDplex online software (https://legendplex.qognit.com/; BioLegend). Plasma levels of CXCL10 were examined using ELISA. Serum levels of IL-1β, IL-6, IL-8, IL-10, TNF-α and sIL-2R and proportions of peripheral immune cells were obtained from routine clinical tests.

### Immunofluorescence chemistry

Liver sections were stained with anti-CD45 (Servicebio, China; #GB113886). The stained sections were then scanned using a Pannoramic DESK tissue slide digital scanner (3DHISTECH, Hungary). Images were captured and visualized using CaseViewer 2.4 digital slide browsing software (3DHISTECH).

### Statistical analysis

Statistical analyses were performed using GraphPad Prism 10.1.2 (GraphPad Software, LLC., San Diego, CA, USA). Data distribution normality was assessed using the Shapiro–Wilk test. Normally distributed parameters were analysed using Student’s *t* test, whereas non-normally distributed data were evaluated using the Mann–Whitney *U* test. Data visualization was performed as follows: Fig. 1 presents results as median with interquartile range (IQR), whereas Fig. 2 displays data using violin plots (with median line and kernel density estimation) supplemented by overlaid individual data points. Relationships between two continuous variables were assessed using Pearson correlation coefficient for parametric data or Spearman rank correlation coefficient for non-parametric data (two-tailed; *P* < 0.05). For in vitro and animal studies, intergroup comparisons were performed using either unpaired two-tailed Student’s *t* tests (for two groups) or one-way ANOVA with Dunnett’s post-test (for multiple groups) or Bonferroni correction (all pairwise comparisons), with data expressed as mean ± SEM.

## Results

### Elevated monocyte-derived itaconate in patients with Still’s disease/macrophage activation syndrome correlates with disease severity

Considering the metabolic reprogramming of the TCA cycle during activation of monocytes and macrophages^18,19^, we performed a targeted metabolomics analysis of TCA cycle and its associated metabolites with serum samples from patients with SD (Fig. 1a). Compared with HCs, patients with SD exhibited elevated levels of pyruvate (2.013-fold), itaconate (2.095-fold) and α-ketoglutarate (1.494-fold), whereas citrate (0.666-fold), aconitate (0.607-fold), isocitrate (0.635-fold) and succinate (0.732-fold) were reduced (Fig. 1a). Notably, serum itaconate levels demonstrated a positive correlation with systemic disease severity scores (Fig. 1b), indicating itaconate as a metabolic biomarker of SD severity. We also observed a positive correlation between serum itaconate levels and peripheral monocyte counts (but not neutrophils or lymphocytes; Fig. 1c, Supplementary Fig. 2a–c). Further, mRNA of *ACOD1* (encoding the itaconate-producing enzyme IRG1) was upregulated in PBMCs of patients with SD (Fig. 1d). RNA-seq analysis of peripheral blood immune cells from patients with SD (*n* = 18, publicly available dataset E-GEAD-397 (ref.^32^)) revealed *ACOD1* expression predominantly in classical (CD14^++^CD16^−^) and non-classical (CD14^+^CD16^+^) monocytes, rather than other immune subsets (Supplementary Fig. 2d). In line with the gene expression, the IRG1 protein level was markedly higher in monocytes of patients with SD (Fig. 1e). We also compared *ACOD1* expression of peripheral blood monocytes in patients with SD with or without MAS (from our publicly available datasets GSE247993 (ref.^15^)), and observed markedly higher expression of *ACOD1* in patients with MAS (Supplementary Fig. 2e). To sum up, these results indicate the implication of the peripheral monocyte-specific *ACOD1*–itaconate axis in SD/MAS progression.

### Association of CXCL10 and CD8^+^ T cells with macrophage activation syndrome

In that patients with SD/MAS had greater expression of *ACOD1* in circulating monocytes than patients without MAS (Supplementary Fig. 2e), we were wondering if there are systemic inflammatory indicators associated with MAS. In the peripheral blood, we first confirmed an elevation of soluble IL-2R (sIL-2R) (Fig. 2a) a common biomarker of MAS and an indicator of T cell over-activation^36,37^. In addition, we observed significantly increased levels of CXCL10 and IL-10 in patients with SD/MAS (Fig. 2b). Consistent with the role of CXCL10 as the most potent chemokine to induce chemotaxis of T cells^38,39^, immunophenotyping of the peripheral blood revealed an elevation in the percentage of CD8^+^ T cells in these patients (Fig. 2c). Further, the plasma CXCL10 correlated positively with serum IL-10 and sIL-2R but negatively with platelet counts (Fig. 2d). Plasma CXCL10 level also correlated with ALT and AST, markers of liver damage commonly associated with SD severity^40,41^ (Fig. 2d). Crucially, plasma CXCL10 level also exhibited a positive correlation with CD8^+^ T cell percentage (Fig. 2e), whereas no correlations were observed between CXCL10 and other immune-cell subsets (data not shown). Collectively, these results indicated a potential CXCL10–CD8^+^ T cell axis in the progression of SD/MAS.

### Activated *Acod1*/itaconate pathway in a murine macrophage activation syndrome model

To investigate the role of itaconate in MAS pathogenesis, we utilized a CpG ODN 1826-induced murine model that recapitulates key clinical features of human MAS^33^. CpG-challenged mice developed a characteristic decrease in the numbers of platelets and red blood cells, and exhibited elevated serum levels of IL-6, IL-18 and TNF-α (Supplementary Fig. 3a, b), accompanied by significant increases in CXCL10, CXCL1 and CCL2 (Supplementary Fig. 3c). Notably, these animals displayed peripheral lymphopenia with compensatory neutrophilia (Supplementary Fig. 3d), mirroring the haematological manifestations observed in patients with SD/MAS.

**Fig. 3:**
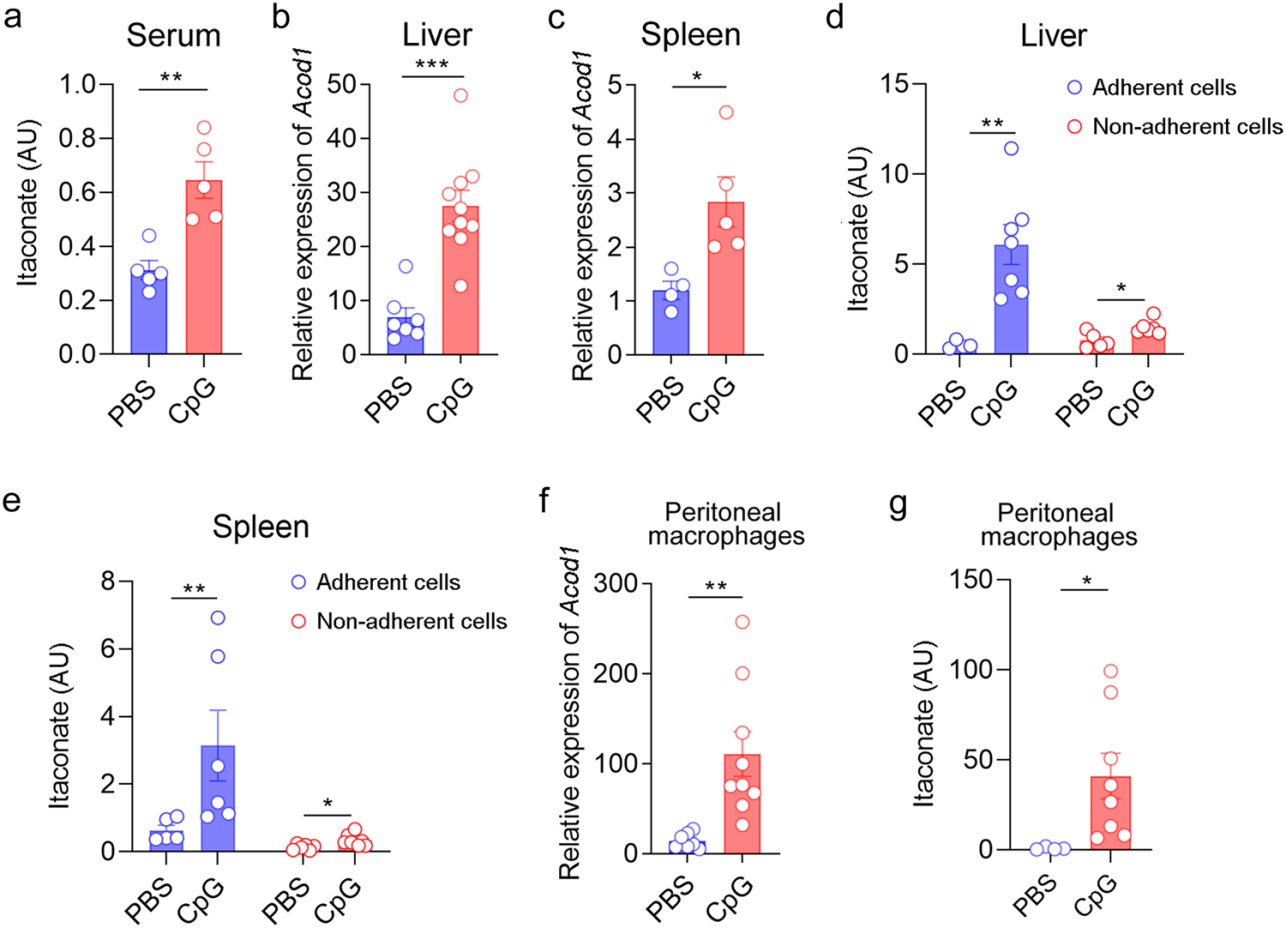
Elevated *Acod1*/itaconate levels in CpG ODN 1826-induced macrophage activation syndrome-like mice. **a**, Serum itaconate levels in PBS- vs CpG-treated mice (*n* = 5) measured by liquid chromatography-mass spectrometry (LC-MS). **b**, **c**, *Acod1* mRNA expression in liver (**b**) or spleen (**c**) of PBS- (*n* = 7/4) vs CpG-treated mice (*n* = 10/5). **d**, **e**, Itaconate levels in liver (**d**) or spleen (**e**) adherent (monocytes/macrophages, isolated by plastic adherence) vs non-adherent cells from PBS- (*n* = 4–6) vs CpG-treated mice (*n* = 6–7) measured by LC-MS. **f**, *Acod1* mRNA expression in peritoneal macrophages of PBS- (*n* = 7) or CpG-treated mice (*n* = 9). **g**, Itaconate levels in peritoneal macrophages of PBS- (*n* = 4) or CpG-treated mice (*n* = 8) measured by LC-MS. The bar plots were presented as mean ± SEM. *P < 0.05; **P < 0.01; ***P < 0.001. AU represents arbitrary units for signal intensity.

Consistent with elevated serum itaconate in patients with SD (Fig. 1a), CpG-challenged mice exhibited increased circulating itaconate (Fig. 3a). Tissue-specific gene-expression analysis revealed upregulations of *Acod1* expression in livers and spleens of CpG-stimulated mice (Fig. 3b, c). In these tissues, intracellular itaconate accumulation was more pronounced in adherent cells (monocytes/macrophages), indicating that they are the main producers of itaconate (Fig. 3d, e). In addition, peritoneal macrophages from CpG-treated mice exhibited *Acod1* induction and intracellular itaconate accumulation (Fig. 3f, g), in line with the conserved itaconate synthesis in monocytic lineage across species.

### *ACOD1*-IRG1-itaconate axis transcriptionally activated by CpG in monocytes/macrophages

**Fig. 4:**
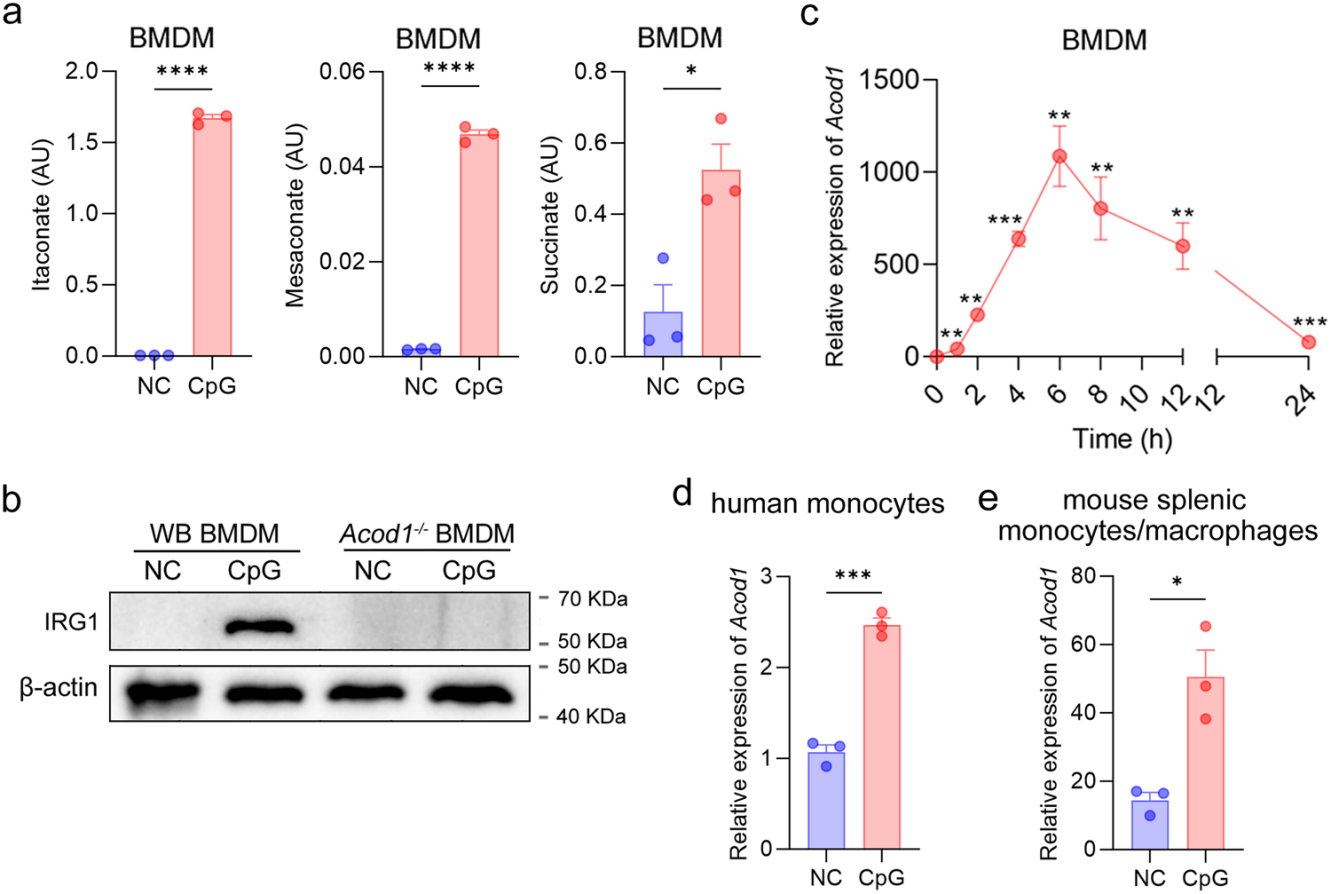
CpG ODN 1826 activates the *ACOD1*–IRG1–itaconate pathway in monocytes/macrophages. **a** Levels of itaconate, mesaconate and succinate in bone marrow-derived macrophages (BMDMs) treated with CpG ODN 1826 (1 μM) for 18 h, measured by gas chromatography/mass spectrometry (GC-MS) (*n* = 3). **b** Western blot analysis of IRG1 (53 kDa) in CpG (1 μM)-treated WT or *Acod1*^*−*^^*/*^^*−*^ BMDMs. β-actin (42 kDa) was the loading control. **c** Time-course of *Acod1* mRNA expression in BMDMs treated with 1 μM CpG (quantitative PCR, *n* = 3). **d**, **e** *ACOD1* mRNA expression in human peripheral blood monocytes (**d**) or mouse spleen monocytes/macrophages (**e**) treated with CpG ODN 1826 (1 μM) for 3 h (*n* = 3). Figures are representative of three independent experiments. The bar plots were presented by mean ± SEM. *P < 0.05; ***P < 0.001. AU represents arbitrary units for signal intensity.

Next, we aimed to elucidate if CpG ODN 1826 has a direct activation on the *ACOD1*–IRG1–itaconate axis. We first analysed intracellular metabolites of CpG-activated mouse BMDMs and observed significant production of itaconate in these activated macrophages but not in control cells (Fig. 4a). Mesaconate, a downstream metabolite of itaconate recently identified by us,^24^ also accumulated by CpG stimulation of BMDMs (Fig. 4a), providing additional evidence of activated itaconate production. The TCA cycle intermediate succinate was significantly elevated in CpG-stimulated BMDMs (Fig. 4a), in line with itaconate-induced inhibition on succinate dehydrogenase activity^20,42^.

At the protein level, CpG clearly induced IRG1 in BMDMs, which was entirely abolished in BMDMs from *Acod1*-deficient mice (Fig. 4b). Further, a time-course analysis of *Acod1* mRNA in CpG-stimulated BMDMs exhibited a prompt activation, which peaked at 6 h before a gradual decline (Fig. 4c). Finally, as evidenced by CpG-induced *ACOD1* upregulation in both human peripheral blood monocytes and murine splenic monocytes/macrophages (Fig. 4d, e), we confirmed the CpG-induced *Acod1* expression not only in macrophages but also in their precursor monocytes.

### Complexity of itaconate-modulated cyto-/chemokine production in CpG-activated monocytes/macrophages

**Fig. 5:**
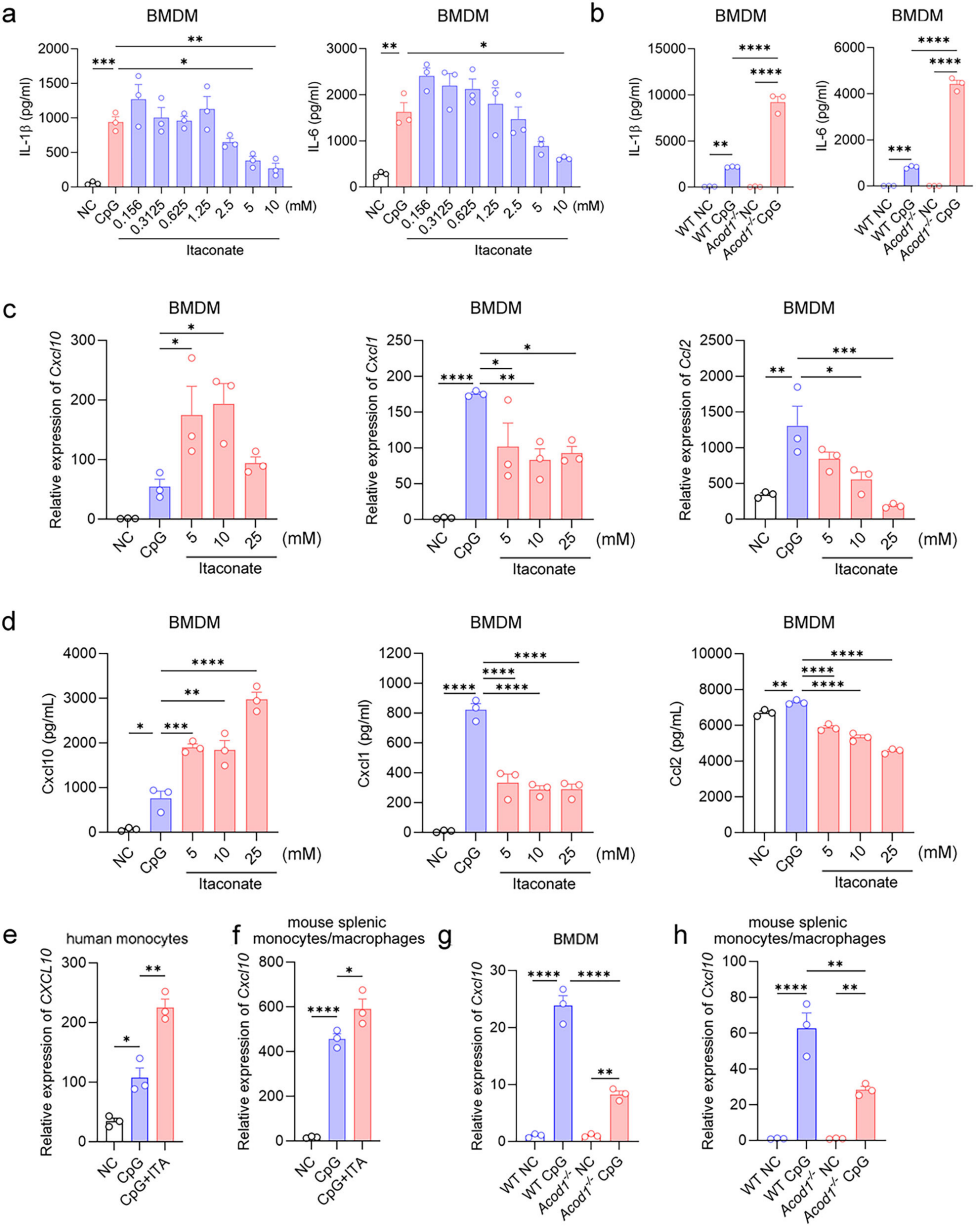
Itaconate differentially modulates CpG ODN 1826-induced cytokine and chemokine production. **a** IL-1β and IL-6 levels in supernatants of bone marrow-derived macrophages (BMDMs) pretreated with itaconate for 2 h (indicated concentrations), followed by CpG (1 μM) stimulation for 6 h (*n* = 3). For measurement of IL-1β, 5 mM ATP was added 30 min prior to supernatant collection (also applicable to other figures exhibiting secreted IL-1β). **b** IL-1β and IL-6 levels in supernatants of WT or *Acod1*^*−/−*^ BMDM after CpG (1 μM, 6 h) stimulation (*n* = 3). **c** Gene-expression analysis of *Cxcl10, Cxcl1* and *Ccl2* in BMDMs after CpG (1 μM, 3 h) stimulation with itaconate pre-treatment (2 h) (*n* = 3). **d** CXCL10, CXCL1 and CCL2 levels in supernatants of BMDMs pretreated with itaconate for 2 h, followed by CpG (1 μM) stimulation for 18 h (*n* = 3). **e**, **f** *CXCL10* mRNA in human peripheral monocytes (**e**) or mouse spleen monocytes/macrophages (**f**) pretreated with itaconate (10 mM, 2 h), followed by CpG (1 μM, 3 h) stimulation (*n* = 3). **g**, **h** *Cxcl10* mRNA in WT or *Acod1*^*−/−*^ BMDMs (**g**) or mouse spleen monocytes/macrophages (**h**) after CpG (1 μM, 3 h) stimulation (*n* = 3). Figures are representative of three independent experiments. The bar plots were presented as mean ± SEM. *P < 0.05; **P < 0.01; ***P < 0.001; ****P < 0.0001.

Considering the well-established immunomodulatory role of itaconate in LPS-stimulated monocytes/macrophages^17,28^, we next aimed to understand if it is the same case in CpG-treated monocytes/macrophages. Although low doses of exogenous itaconate tend to increase CpG-stimulated IL-6 secretion, itaconate at higher doses reduced IL-1β and IL-6 secretion from CpG-stimulated BMDMs (Fig. 5a). Consistently, itaconate decreased CpG-induced *IL-1β* expression in human monocytes (Supplementary Fig. 4a). To elucidate the role of endogenous itaconate, we employed *Acod1*-KO mouse strain. Genetic ablation of *Acod1* exacerbated CpG-induced IL-1β/IL-6 gene expression and protein release in BMDMs and splenic monocytes/macrophages (Fig. 5b, Supplementary Fig. 4b, c), indicative of the anti-inflammatory activity of endogenous itaconate. In accordance, *Acod1* deficiency also amplified *Il1b/Il6* expression in LPS-stimulated BMDMs (Supplementary Fig. 4d). 4-OI and DMI, two representative chemical derivatives of itaconate, also suppressed *IL-1β/IL-6* expression in CpG-stimulated mouse BMDMs and human monocytes (Supplementary Fig. 4e, f).

Given the implication of CXCL10 in the development of MAS (Fig. 2b), we were wondering about the role of itaconate in CpG-induced chemokine production. The addition of exogenous itaconate significantly reduced both the expression and secretion of CCL2 and CXCL1 from CpG-stimulated BMDMs (Fig. 5c, d). In contrast, exogenous itaconate augmented CpG-stimulated production of CXCL10, both at the gene-expression and at the secreted-protein level. This effect was contrary to the suppression of IL-1β and IL-6 by itaconate (Fig. 5a), as well as the suppression of CXCL10 expression by 4-OI and DMI (Supplementary Fig. 4g), which is in accordance with our previous finding that the underivatized form of itaconate enhances LPS-induced CXCL10 production in mouse macrophages (also in Supplementary Fig. 4h)^24^. Further, augmentation of CXCL10 expression by itaconate but not its derivatives was also observed in CpG-stimulated human and mouse monocytes (Fig. 5e, f, and Supplementary Fig. 4i). Finally, *Acod1* deficiency reduced CXCL10 expression from CpG-treated mouse BMDMs and monocytes (Fig. 5g, h), indicative of the unanimous action of endogenous and exogenous itaconate to enhance CXCL10 production. The immunomodulatory effects of itaconate on cytokine and chemokine expression were further confirmed by RNA-sequencing analysis: itaconate promoted *Cxcl10, Cxcl3* and *Cxcl5* expression, while reducing *IL1a, IL-1b* and *IL-12b* expression in CpG-induced BMDMs (Supplementary Fig. 4j).

It is well established that the IFN-γ–CXCL10 axis is important in the pathogenesis of MAS^43^. Although we confirmed IFN-γ-induced CXCL10 expression in macrophages (Supplementary Fig. 4k), itaconate had no synergistic effects on CXCL10 expression as it did in CpG-induced CXCL10 expression. As IL-10 was also elevated in patients with SD with MAS (Fig. 2a), we additionally examined the potential implication of itaconate for IL-10 production in CpG-stimulated macrophages. Unlike the phenomenon observed for CXCL10, itaconate significantly suppressed IL-10 secretion from these cells (Supplementary Fig. 4l), which excluded the possibility of an itaconate–IL-10 axis in MAS.

To understand the clinical relevance of the ACOD1–CXCL10 axis, we analysed recent transcriptomic datasets from patient-derived monocytes. In SD monocytes (GSE247993)^15^, we observed a significant positive correlation between *ACOD1* and *CXCL10* expression levels (Supplementary Fig. 5a), whereas no correlation is observed between *ACOD1* and *IL-10*. In another independent dataset (E-GEAD-397)^32^, we confirmed this correlation in both classical and non-classical monocytes from patients with SD (Supplementary Fig. 5b). Notably, such correlations between ACOD1 and CXCL10 expression in monocytes were more pronounced in macrophage activation-driven SD (*r* = 0.7228 and *r* = 0.6007 in classical and non-classical monocytes, respectively) than in adaptive immunity-dominated systemic lupus erythematosus (*r* = 0.4621 and *r* = 0.4129 in classical and non-classical monocytes, respectively; *P* < 0.01 for both comparisons) (Supplementary Fig. 5c). This disease-specific pattern might suggest preferential coupling of the ACOD1–itaconate axis with CXCL10 production in macrophage-driven pathologies.

### ACOD1 dictates systemic disease manifestations and hepatic immunopathology

**Fig. 6:**
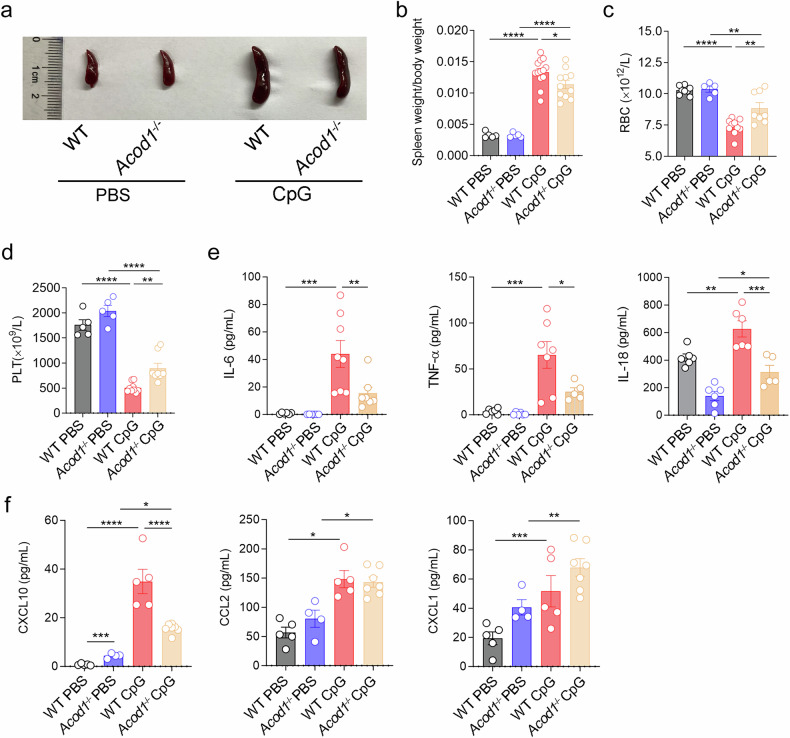
*ACOD1*-deficiency alleviates disease manifestations of CpG ODN 1826-induced macrophage activation syndrome-like mice. **a** Representative images of spleens from CpG-stimulated wild type (WT) or *Acod1*^*−/*^^*−*^ mice. **b** Spleen index (spleen weight/body weight) in WT (PBS, *n* = 5; CpG, *n* = 13) and *Acod1*^*−/−*^ (PBS, *n* = 5; CpG, *n* = 11) mice. **c**, **d** RBC (**c**) and PLT (**d**) counts in whole blood from WT (PBS, *n* = 5–8; CpG, *n* = 9–10) and *Acod1*^*−/−*^ (PBS, *n* = 5; CpG, *n* = 8) mice. **e**, **f** Serum levels of IL-6, TNF-α, IL-18 (**e**) and CXCL10, CCL2, CXCL1 (**f**) in WT (PBS, *n* = 5–6; CpG, *n* = 5–8) and *Acod1*^*−/−*^ (PBS, *n* = 4–6; CpG, *n* = 5–7) mice. The bar plots were presented as mean ± SEM. *P < 0.05; **P < 0.01; ***P < 0.001; ****P < 0.0001. RBC, red blood cell; PLT, platelet.

To determine the pathological relevance of the immunomodulatory effects of itaconate observed in vitro, we employed the *Acod1*-deficient mouse strain (*Acod1*^*−/−*^) in the CpG ODN 1826-induced MAS model. Genetic ablation of *Acod1* significantly attenuated systemic disease manifestations, evidenced by reduced splenomegaly (Fig. 6a, b) and ameliorated anaemia/thrombocytopenia (Fig. 6c, d). Profiling of circulating cytokines and chemokines revealed marked reduction of CpG-induced IL-6, TNF-α and IL-18 production in *Acod1*^*−/−*^ mice, compared with WT mice (Fig. 6e). In parallel, *Acod1* deficiency selectively reduced CXCL10 but not CCL2/CXCL1 secretion (Fig. 6f), mirroring the specific itaconate–CXCL10 axis identified in cultured macrophages and monocytes (Fig. 5c–f).

**Fig. 7:**
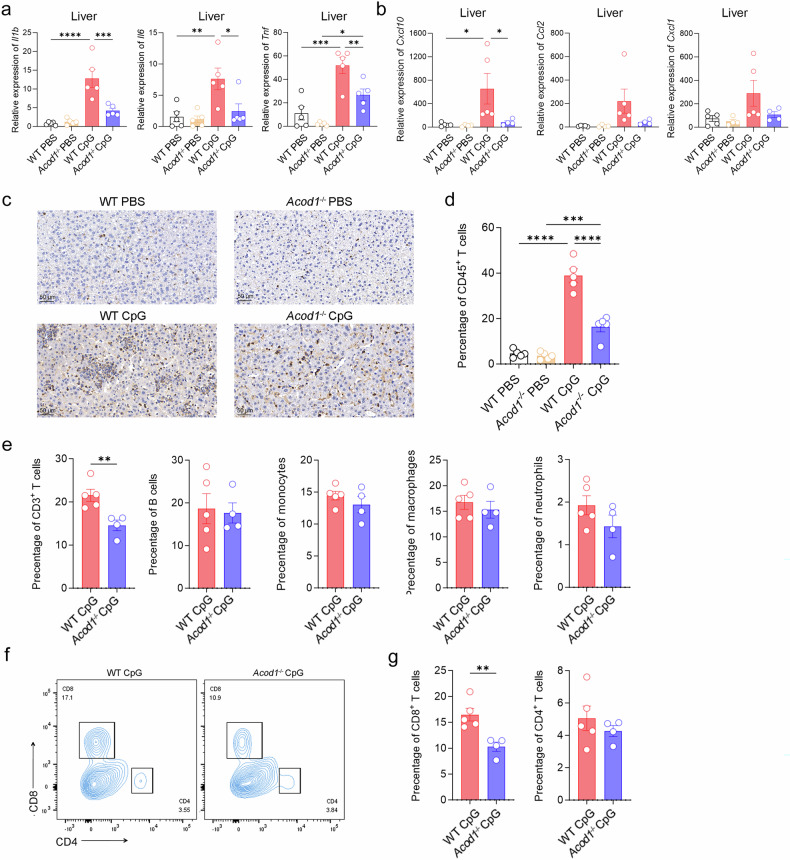
*ACOD1* deficiency mitigates hepatic inflammation and CD8^+^ T cell infiltration in CpG ODN 1826-induced mice. **a**, **b,** Quantitative PCR analysis of *Il1b, Il6, Tnf* (**a**) and *Cxcl10, Ccl2, Cxcl1* (**b**) mRNA in livers of wild type (WT) and *Acod1*^*−/−*^ (PBS/CpG, *n* = 4–5) mice. **c** Immunohistochemical staining for CD45 in livers of WT and *Acod1*^*−/−*^mice (*n* = 5) at ×200 magnification (scale bar = 50 μm). **d** Percentage of CD45^+^ cells by flow cytometric analysis of livers from WT (PBS/CpG, *n* = 5) and *Acod1*^*−/−*^ (PBS/CpG, *n* = 5) mice. **e** Percentages of CD3^+^ T, B, monocytes, macrophages and neutrophils by flow cytometric analysis of livers from CpG-treated WT (*n* = 5) and *Acod1*^*−/−*^ mice (*n* = 4). **f** Representative flow cytometric plots of hepatic T cell subsets. **g** Percentages of CD3^+^CD8^+^ and CD3^+^CD4^+^ T cells in total hepatic cells (*n* = 4–5). The bar plots were presented by mean ± SEM. *P < 0.05; **P < 0.01; ***P < 0.001; ****P < 0.0001.

The liver inflammation is associated with pathogenesis of MAS, and liver damage is indicative of severe SD including MAS^40,41^. Our results also suggest a correlation between CXCL10 level and liver damage in patients with SD with MAS (Fig. 2d). In the CpG-induced MAS model, hepatic gene-expression analysis exhibited significantly lower expression of *Il1b, Il6, Tnf* and *Cxcl10* in *Acod1*-deficient mice (Fig. 7a, b), manifesting ameliorated hepatic inflammation. Immunohistochemical and flow cytometric analysis consistently revealed a reduction in CpG-triggered CD45^+^ leukocyte infiltration into the livers of *Acod1*-deficient mice compared with WT livers (Fig. 7c, d). Further immunophenotyping analysis revealed a reduced proportion of hepatic T cells in *Acod1*-deficient mice, which was not seen in B cells, macrophages, monocytes and neutrophils (Fig. 7e). This is in line with the itaconate–CXCL10 axis observed in this study, as CXCL10 is known for its potent chemotactic effect on T cells. At last, we identified CD8^+^ T cells, rather than CD4^+^ T cells, as the T cell subset that is reduced by *Acod1* deficiency (Fig. 7f, g). Collectively, these results indicate that endogenous itaconate is a predominant contributor to experimental MAS progression via selective licencing of hepatic CD8^+^ T cell accumulation.

## Discussion

Our study unveils a previously unrecognized metabolic–immune crosstalk in SD/MAS, positioning monocyte/macrophage-derived itaconate as a dual regulator that paradoxically suppresses canonical pro-inflammatory cytokines while licencing CXCL10-mediated CD8^+^ T cell-driven tissue injury. The elevation of serum itaconate in patients with SD and its correlation with monocyte counts and disease severity (Fig. 1) suggest its potential involvement in disease-associated monocyte/macrophage activation, inflammatory responses and pathological damage. This is consolidated by the identification of the *Acod1*–itaconate axis in monocytes and macrophages in both a mouse model of MAS and in vitro cell cultures (Figs. 3, 4). Specific enhancement of CXCL10 production by itaconate in monocytes and macrophages (Fig. 5) is reminiscent of elevated plasma CXCL10 level in patients with MAS and further the correlation between CXCL10 and CD8^+^ T cells in these patients (Fig. 2). In the MAS mouse model, endogenous itaconate causes disease manifestations and, notably, hepatic inflammation as well as the CXCL10–CD8^+^ T cell axis in the liver (Figs. 6, 7).

Immune cells undergo rapid and extensive metabolic reprogramming upon activation. Certain metabolites accumulate during this process and function as immune regulators, hereby termed immunometabolites^19^. Myeloid cell-specific itaconate is presumably the most famous immunometabolite, owing to its enormous intracellular accumulation (up to 7.5 mM in activated macrophages) and its potent anti-inflammatory effects^28,44^. Itaconate is synthesized from the TCA cycle via the inducible enzyme ACOD1, also known as IRG1. Expression of this enzyme is induced by diverse stimuli — including ligands to multiple TLRs, and various pro-inflammatory cytokines in different conditions including infection, inflammation, autoimmune, cancers and metabolic complications^27,45^. In SD, the existence of various ligands to TLRs, excessive pro-inflammatory cytokines and pathogen- and damage-associated molecular patterns creates an ideal environmental cue for the induction of ACOD1 expression. In line with this, our clinical evidence showed elevated ACOD1 expression in monocytes of patients with SD, particularly in patients with MAS (Fig. 1e and Supplementary Fig. 2e). Notably, clinical observations of elevated cytomegalovirus (CMV) DNA copies and anti-CMV antibodies in patients with active SD suggest that CMV-derived CpG motifs engage TLR9 to exacerbate disease progression^5^. Based on this, CpG ODN 1826 — a phosphorothioate-modified oligonucleotide with enhanced immunostimulatory potency — is widely used to establish MAS models to mimic human disease^33^. Although TLR9 activation-induced mouse *Acod1* expression has been reported in myeloid cells^46,47^, we were to our knowledge the first to validate this metabolic pathway by metabolomics analysis — CpG-stimulated mouse macrophages accumulate itaconate and its downstream metabolite mesaconate (Fig. 4a). Our consistent results validating the ACOD1–itaconate axis in human and mouse cells also exhibit evolutionary conservativeness across species, warranting the effectiveness of investigating this axis in a mouse MAS model.

As SD is a type of hyperferritinaemic syndrome and ferritin serves as a key biomarker for SD/MAS^48^, we further analysed the correlation between itaconate and ferritin in our cohort of patients with SD (*n* = 20). Although hyperferritinaemia was observed as a characteristic of the disease (1690.0 ± 1542.0 ng/ml, in contrast to the clinical reference level of serum ferritin for HD of < 306.8 ng/ml; Supplementary Table 1), we did not observe a correlation between serum ferritin and itaconate levels (*r* = −0.2843, *P* = 0.2244; Supplementary Fig. 6a). Then, we performed analysis of ferritin expression in our CpG-induced MAS model. In livers, there was no difference in the expression of ferritin between the CpG-treated and PBS groups (Supplementary Fig. 6b), whereas in spleens CpG treatment led to significant ferritin upregulation compared with controls (Supplementary Fig. 6c). The expression of ferritin was affected by *Acod1* deficiency neither in PBS control nor CpG-treated mice (Supplementary Fig. 6c). Therefore, our results support ferritin as a crucial biomarker in SD/MAS, whereas the immunomodulatory effects of itaconate are likely to operate independently of ferritin.

The anti-inflammatory role of itaconate has been well documented in various inflammatory settings and disease models^20–22^. Notably, the initial studies employed derivatives of itaconate, aiming to achieve better permeability across the cell membrane. Following studies revealed that the underivatized form of itaconate is indeed immunomodulatory, rather than simply anti-inflammatory^23–26^. Specifically, although confirming the suppression on various pro-inflammatory cytokines, we observed a boosting effect of itaconate and its downstream product mesaconate on LPS-stimulated CXCL10 secretion in mouse macrophages^24^. Other studies exhibited an enhanced production of IFN-β by itaconate in both human and mouse macrophages^23–26^. As CXCL10 is a chemokine downstream of the IFN-β signaling pathway, it is suggestive of an itaconate–IFN-β–CXCL10 axis in macrophages. In this current study, we extended this dualistic paradigm to TLR9-driven inflammation. Our in vitro data establish itaconate as immunomodulatory rather than a broad-spectrum anti-inflammatory agent in CpG-activated macrophages — itaconate suppresses a set of canonical pro-inflammatory cytokines (e.g. *IL1a, IL-1b* and *IL-12b*) and chemokines (e.g. *Ccl4, Ccl5* and *Ccl6*), but amplifies *Cxcl10, Cxcl3* and *Cxcl5* (Fig. 5 and Supplementary Fig. 4j). This was in line with our previous studies using LPS-stimulated macrophages, where itaconate reduced IL-6 and IL-12 whereas it boosted a few other cytokines and chemokines, including CXCL10 (ref.^24^). This consistency across different TLR stimuli (TLR4 and TLR9) highlights a fundamental and reproducible immunomodulatory signature of underivatized itaconate. Interestingly, the itaconate derivatives such as 4-OI and DMI universally inhibited production of these cytokines and chemokines, suggesting divergent mechanisms between the original itaconate and its synthetic derivatives. In CpG-activated monocytes/macrophages, underivatized itaconate selectively suppressed IL-1β, IL-6, CCL2 and CXCL1 but amplified CXCL10 (Fig. 5a–f), whereas 4-OI/DMI inhibited all the cytokines and chemokines tested, including CXCL10 (Supplementary Fig. 4e–g, i).

CXCL10 — a pleiotropic chemokine mediating recruitment of CXCR3^+^ T cells, including CD8^+^ T cells^38,39^ — is elevated across autoimmune diseases such as rheumatoid arthritis and lupus, where it triggers effector T cell trafficking and interferon-driven pathology^49,50^. In SD, although prior studies noted elevated CXCL10 levels^11^, we demonstrate that plasma CXCL10 distinguishes MAS from non-MAS SD, positioning it as a stratification biomarker^15^. We identified monocytes/macrophages as likely cellular sources of this pathogenic CXCL10, as evidenced by marked upregulation of CXCL10 in monocytes from patients with SD/MAS in our transcriptomic analysis^15^ and robust CXCL10 production by human monocytes/macrophages stimulated with either LPS or CpG in vitro (Fig. 5c–f, Supplementary Fig. 4h). Building on these lines of evidence, further clinical analysis demonstrated that patients with SD/MAS exhibit increased proportions of circulating CD8^+^ T cells, which correlated positively with plasma CXCL10 levels (Fig. 2e). Studies in autoimmune hepatitis have revealed CD8^+^ T cell-driven hepatocyte pyroptosis via granzyme B and perforin-1, directly linking cytotoxic T cell activity to parenchymal damage^51^. Although the exact mechanisms of CD8^+^ T cells in SD require further investigation, the clinical observations that patients with SD with hepatic involvement exhibit significantly elevated proportions of circulating CD8^+^ T cells compared with those without liver injury, and that these CD8^+^ T cell levels correlate positively with serum AST levels, suggest a causal role in SD-associated liver damage^40^. Critically, these clinical patterns were recapitulated in our CpG-induced MAS model, where *Acod1* deficiency significantly reduced serum CXCL10 levels and hepatic CD8^+^ T cell infiltration without affecting serum CCL2/CXCL1 concentrations or myeloid-cell recruitment (Fig. 6f, and Fig. 7e, f), ultimately leading to amelioration of MAS-associated clinical symptoms and systemic inflammation (Fig. 6). CXCL10 is the key chemokine recruiting pathogenic CD8^+^ T cells into the liver, which are major drivers of tissue injury and systemic inflammation in this model. Therefore, the absence of itaconate in *Acod1*^*−/−*^ mice disrupts this critical CXCL10–CD8^+^ T cell axis and in turn reduces CD8^+^ T cell-associated tissue damage and inflammatory responses, including production of IL-6, TNF-α and IL-18 (Fig. 6e). The observed overall effects in KO mice exhibited an attenuation of systemic inflammation, along with ameliorated symptoms (Fig. 6), indicating that the CD8^+^ T cells dictate the inflammatory status by outweighing the seemingly distinct cytokine profile in macrophages. This also exemplifies the importance of the in vivo disease context when conceptualizing therapeutic targets with pleiotropic effects, such as, but not limited to, the ACOD1–itaconate axis.

IFN-γ is another canonical inducer of CXCL10, and the IFN-γ–CXCL10 axis plays critical roles in MAS^43^. We confirmed IFN-γ-induced CXCL10 expression in mouse macrophages (Supplementary Fig. 4k), although we did not observe the same synergistic effect of itaconate as we saw from CpG-induced CXCL10 expression (Figs. 5c–f). Similarly, *Acod1* knockout does not affect IFN-γ-induced CXCL10 expression^52^. Thus, it seems that itaconate does not influence the IFN-γ-induced CXCL10 pathway but rather selectively promotes the TLR9 (CpG)-driven CXCL10 pathway in our model (Fig. 5). These results indicate that itaconate is not a universal rheostat for CXCL10 expression but is more like a pathway-specific modulator. The selective amplification of the TLR9-driven CXCL10 pathway reveals a novel, immunometabolic layer to the pathogenesis of hyperinflammation models like MAS, which operates in parallel to the well-established IFN-γ pathway. This paradigm enhances our mechanistic understanding by suggesting that distinct triggers (e.g. viral DNA via TLR9, and T cell-derived IFN-γ) can engage complementary pathways to drive a common pathogenic outcome (CXCL10-mediated CD8^+^ T cell tissue injury).

Our findings revise the conventional therapeutic paradigm of utilizing itaconate as a universal anti-inflammatory agent. Although itaconate suppresses canonical pro-inflammatory cytokines (e.g. IL-1β, IL-6) in both LPS- and CpG-stimulated macrophages (Fig. 5a, b, and Supplementary Fig. 4a–d), its dichotomous regulation of chemokines — amplifying CXCL10 while suppressing CCL2/CXCL1 — reveals a context-dependent immunomodulatory spectrum. The therapeutic implication of itaconate-driven CXCL10 amplification is fundamentally determined by the pathogenic context. In our model of MAS, a condition where pathology is critically driven by CD8^+^ T cell infiltration^53^, the dominant effect of itaconate is the licencing of pathogenic immunity. This is demonstrated by the *Acod1* knockout phenotype, where the amelioration of disease — including reduced hepatic CD8^+^ T cell influx and systemic inflammation — is primarily attributable to the loss of CXCL10 amplification (Figs. 6, 7). Therefore, the key therapeutic implication is that the ACOD1–itaconate axis presents a rational target for T cell-driven hyperinflammatory diseases like MAS. The benefit of interrupting the core pathogenic axis CXCL10–CD8^+^ T cells outweighs the potential loss of the ancillary anti-inflammatory effects of itaconate in this specific context. This suggests that patients with high CXCL10 levels, indicative of active T cell recruitment, might be the ideal candidates for such an intervention. This dichotomy underscores that targeting immunometabolic pathways such as the ACOD1–itaconate axis requires biomarker-driven patient stratification.

Notably, our recent RNA-seq data also revealed that itaconate upregulates *Il23a* and *Il17ra* in LPS-stimulated macrophages^24^, suggesting its broader role in licensing Th17-mediated pathology, which is also implicated in the pathogenesis of SD^1^. These observations underscore the risks of unidirectional itaconate modulation. For instance, systemic itaconate supplementation, though anti-inflammatory in sepsis^24^, could exacerbate tissue-specific immunopathology in chronic inflammation by amplifying responses mediated by cytotoxic T cell or Th17 responses. Conversely, broad ACOD1 inhibition might compromise the beneficial effects of itaconate to suppress pro-inflammatory cytokines. To resolve this dilemma, we propose temporally refined strategies: pharmacologically boosting the ACOD1–itaconate pathway during acute hyperinflammation (to suppress pro-inflammatory cytokines) while selectively inhibiting this pathway in chronic phases (to prevent cytokine/chemokine-driven damage from CD8^+^/Th17^+^ T cells). This conceptual framework of temporal modulation suggests that the therapeutic goal may shift from suppressing hyperinflammation acutely to preventing chronic, chemokine-driven tissue damage later in the disease course. Therefore, the targetability of the ACOD1–itaconate–CXCL10 axis mandates a context-specific application, and our current study demonstrates that MAS is representative of such a disease context.

Several limitations merit further investigation. First, although the CpG-induced MAS model effectively recapitulates key inflammatory and haematological features of human MAS, including cytopenias, hepatosplenomegaly and hyperinflammation, it cannot fully mirror the complex immunogenetic background of human SD. Specifically, our murine model lacks the intricate HLA associations and other genetic factors that contribute to disease susceptibility and progression in human SD^2^, thereby limiting direct translation of immunogenetic findings. Furthermore, the acute, monophasic nature of this model does not adequately capture the chronic, relapsing–remitting disease course characteristic of human SD. Additionally, the modest sample size of our clinical cohort precludes subgroup analyses of treatment-naïve versus refractory MAS. Last, although our study identified itaconate as a positive regulator of CXCL10 in the context of SD, the exact transcriptional mechanism awaits further elucidation.

In conclusion, our work discovered an itaconate-mediated metabolic–immune crosstalk in SD/MAS. By extending the immunometabolic role of itaconate to bridge innate immune hyperactivation and adaptive cytotoxicity, this study offers novel mechanistic insights into the pathogenesis of MAS and showcases the itaconate–CXCL10 axis as a potential therapeutic target for immunometabolic intervention.

## Supplementary information

Supplementary Information

## Data Availability

The data that support these findings of the study are available upon request from the corresponding authors.
