# Supplementary material for: Identification of a metabolic–immune crosstalk in Still’s disease: monocyte/macrophage-derived immunometabolite itaconate dictates hepatic immunopathology via the CXCL10–CD8 T cell axis
Source: Exp Mol Med. 2026 Jun 5;58(6):1927–39. doi: 10.1038/s12276-026-01751-x (PMC13324752; doi:10.1038/s12276-026-01751-x)
Supplement: Supplementary file 1 — Supplementary Information [file 12276_2026_1751_MOESM1_ESM.pdf]

Identification of a metabolic-immune crosstalk in Still's disease: monocyte/macrophage-derived immunometabolite itaconate dictates hepatic immunopathology via CXCL10-CD8 T-cell axis

Junna Ye, Fan Wang, Zhuochao Zhou, Jinchao Jia, Ziang Wu, Yijun You, Nadiem Atiq, Yutong Su, Huihui Chi, Jianfen Meng, Mengyan Wang, Yuning Ma, Shirin Hosseini, Martin Korte, Jialin Teng, Chengde Yang, Karsten Hiller, Qiongyi Hu, Wei He and Yue Sun

Supplementary Tables

Supplementary Table 1. Clinical characterization of patients at the time of enrollment

	SD (*n* = 20)
Age(year)	46.9 ± 17.0
Gender(F/M)	15/5
Duration(month)	14.1 ± 19.5
Clinical features	
Fever	19 (95.0)
Sore throat	18 (90.0)
Skin rash	20 (100.0)
Lymphadenopathy	16 (80.0)
Splenomegaly	5 (25.0)
Hepatomegaly	1 (5.0)
Pericarditis	4 (20.0)
Pleuritis	6 (30.0)
Pneumonia	8 (40.0)
Myalgia	9 (45.0)
Arthralgia	19 (95.0)
Arthritis	12 (60.0)
Systemic score	7.1 ± 0.9
Laboratory markers	
Hemoglobin, g/L	117.5 ± 18.5
Leukocyte, ×10^9/L	17.8 ± 4.9
Platelet, ×10^9/L	286.8 ± 90.4
ESR, mm/h	75.3 ± 27.2
CRP, mg/L	95.3 ± 70.2
ALT, U/L	49.6 ± 24.0
AST, U/L	81.7 ± 73.8
Ferritin, ng/mL	1690.0 ± 1542.0
ANA positivity	2 (10.0)
RF positivity	2 (10.0)

All values are presented as n (percentage) or mean ± SD.

SD, Still's disease; *HC*, healthy control subjects; *ESR*, erythrocyte sedimentation rate; *CRP*, C-reactive protein; *AST*, aspartate transaminase; *ALT*, alanine transaminase; *ANA*, anti-nuclear antibody; *RF*, rheumatoid factors.

Supplementary Table 2. Specific primers used in real-time PCR

Name	Species	Gene ID	Primer	Sequence(5’-3’)
ACOD1	human	730249	FW	GCTGCTTTTGTGAACGGTGT
			RV	CTCACCTGTGGCCTGTTGAT
GAPDH	human	2597	FW	GGAGCGAGATCCCTCCAAAAT
			RV	GGCTGTTGTCATACTTCTCATGG
CXCL10	human	3627	FW	GTGGCATTCAAGGAGTACCTC
			RV	TGATGGCCTTCGATTCTGGATT
IL1B	human	3553	FW	ATGATGGCTTATTACAGTGGCAA
			RV	GTCGGAGATTCGTAGCTGGA
IL6	human	3569	FW	ACTCACCTCTTCAGAACGAATTG
			RV	CCATCTTTGGAAGGTTCAGGTTG
Actb	mouse	11461	FW	GGCTGTATTCCCCTCCATCG
			RV	CCAGTTGGTAACAATGCCATGT
Il1b	mouse	16176	FW	GCAACTGTTCCTGAACTCAACT
			RV	ATCTTTTGGGGTCCGTCAACT
Il6	mouse	16193	FW	TAGTCCTTCCTACCCCAATTTCC
			RV	TTGGTCCTTAGCCACTCCTTC
Tnf	mouse	21926	FW	CCCTCACACTCAGATCATCTTCT
			RV	GCTACGACGTGGGCTACAG
Acod1	mouse	16365	FW	TGGTGTTGCTGTTCACTCCA
			RV	TCGGGGGAGTAGTTGGCATA
Ccl2	mouse	20296	FW	TTAAAAACCTGGATCGGAACCAA
			RV	GCATTAGCTTCAGATTTACGGGT
Cxcl10	mouse	15945	FW	CCAAGTGCTGCCGTCATTTTC
			RV	GGCTCGCAGGGATGATTTCAA
Cxcl1	mouse	14825	FW	CTGGGATTCACCTCAAGAACATC
			RV	CAGGGTCAAGGCAAGCCTC
Ftl1	mouse	14325	FW	CCATCTGACCAACCTCCGC
			RV	CGCTCAAAGAGATACTCGCC

Supplementary figures

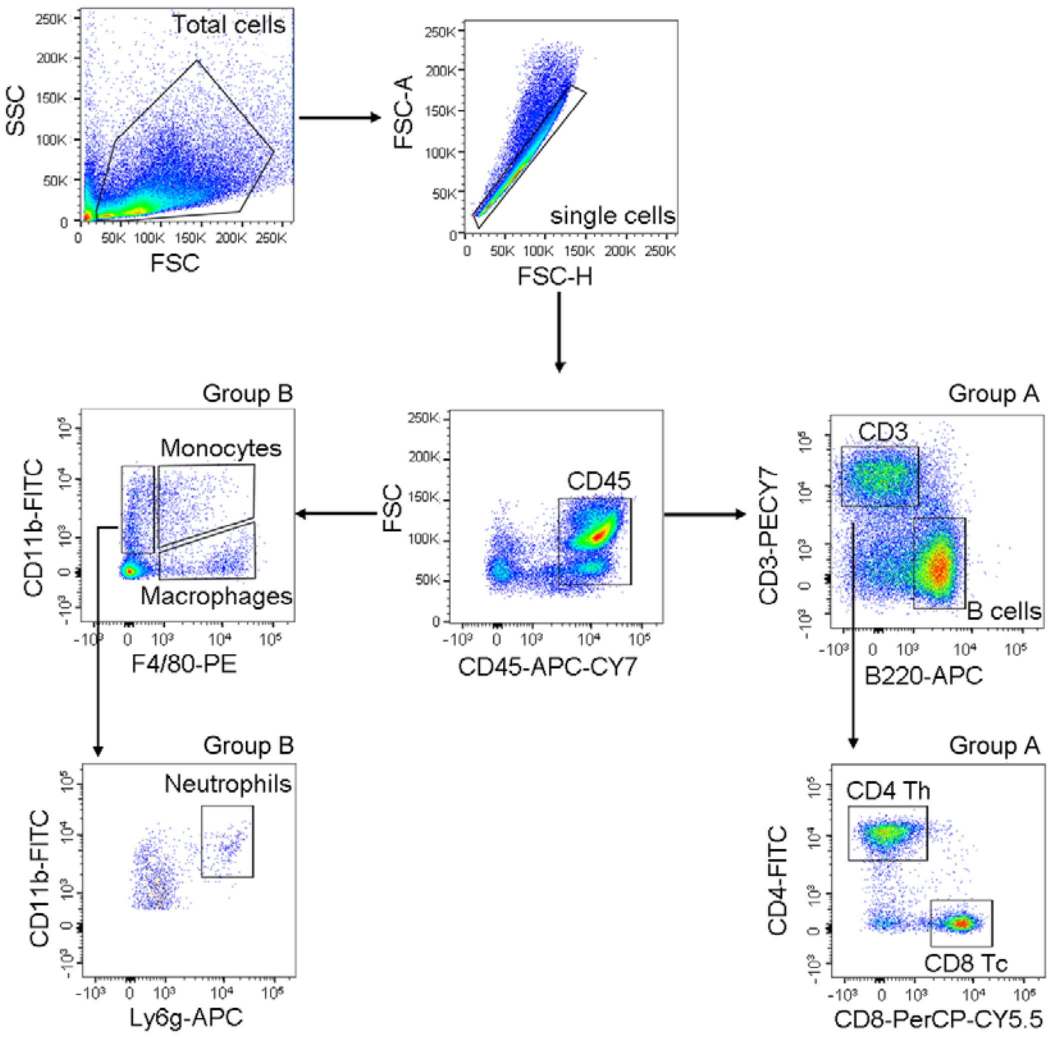

Supplementary Fig. 1. The gating strategy for flow cytometry.

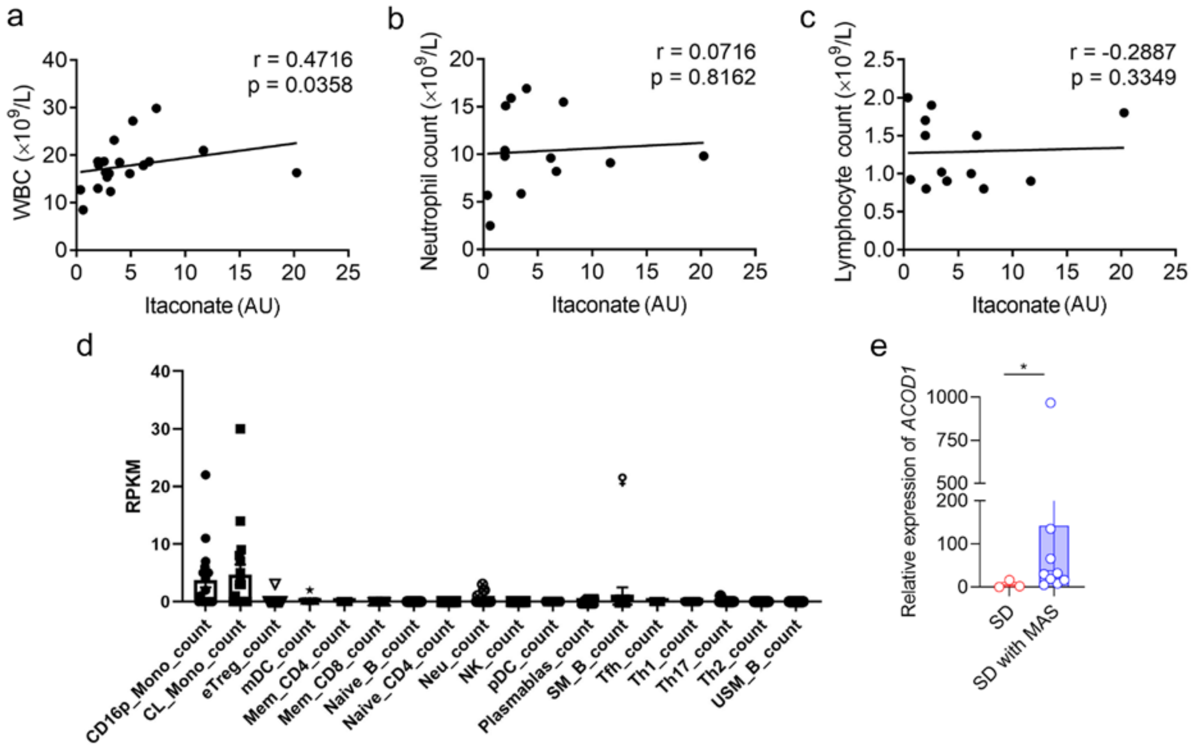

Supplementary Fig. 2 Correlation between serum itaconate levels and immune cell counts in SD patients and *ACOD1* expression in SD/MAS patients. (a-c) Correlations between serum itaconate levels and counts of WBC (a), neutrophil (b), or lymphocyte (c) in active SD patients (n = 20). (d) *ACOD1* mRNA expression in distinct immune cell subsets from whole blood of SD patients (n = 18; cohort from recently published dataset E-GEAD-397). (e) *ACOD1* expression in SD patients without (n = 3) vs. with MAS (n = 9) based on bulk RNA-seq analysis (using recently published dataset GSE247993). The bars were presented by mean ± s.e.m. * $p < 0.05$. WBC, White Blood Cell.

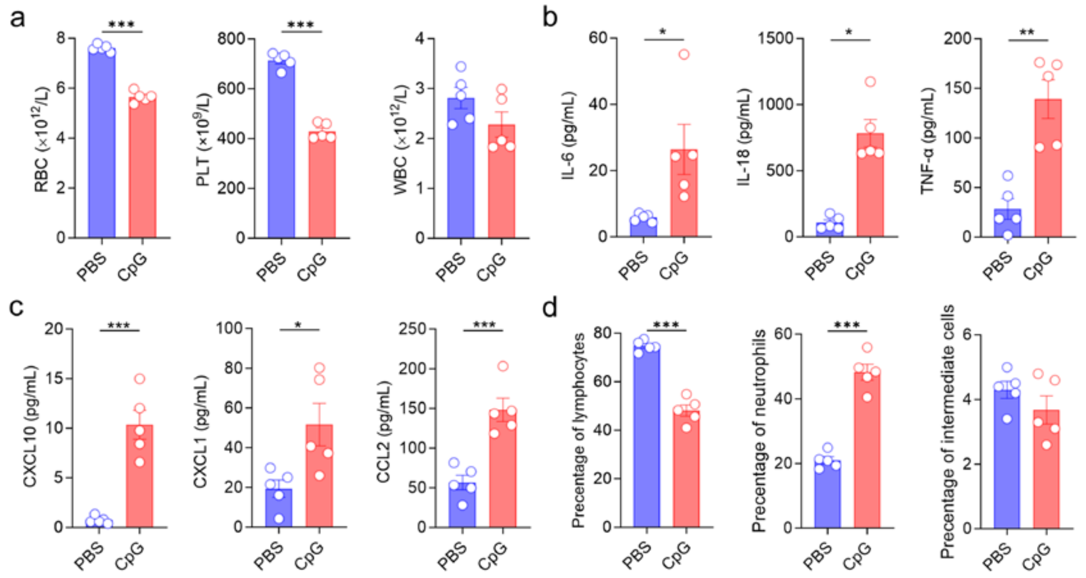

Supplementary Fig. 3 Immunophenotypes of CpG ODN 1826-induced MAS-like mice. (a) RBC, PLT, and WBC counts in whole blood from PBS- or CpG-treated mice (n = 5). (b) Serum levels of IL-6, IL-18, and TNF-α in PBS- vs. CpG-treated mice (n = 5). (c) Serum levels of CXCL10, CXCL1, and CCL2 in PBS- vs. CpG-treated mice (n = 5). (d) Percentages of lymphocytes, neutrophils, and intermediate cells (predominantly monocytes) in whole blood from PBS- vs. CpG-treated mice (n = 5). The bar plots were presented by mean ± s.e.m. * $p < 0.05$; ** $p < 0.01$; *** $p < 0.001$. RBC, Red Blood Cell; PLT, Platelet; WBC, White Blood Cell.

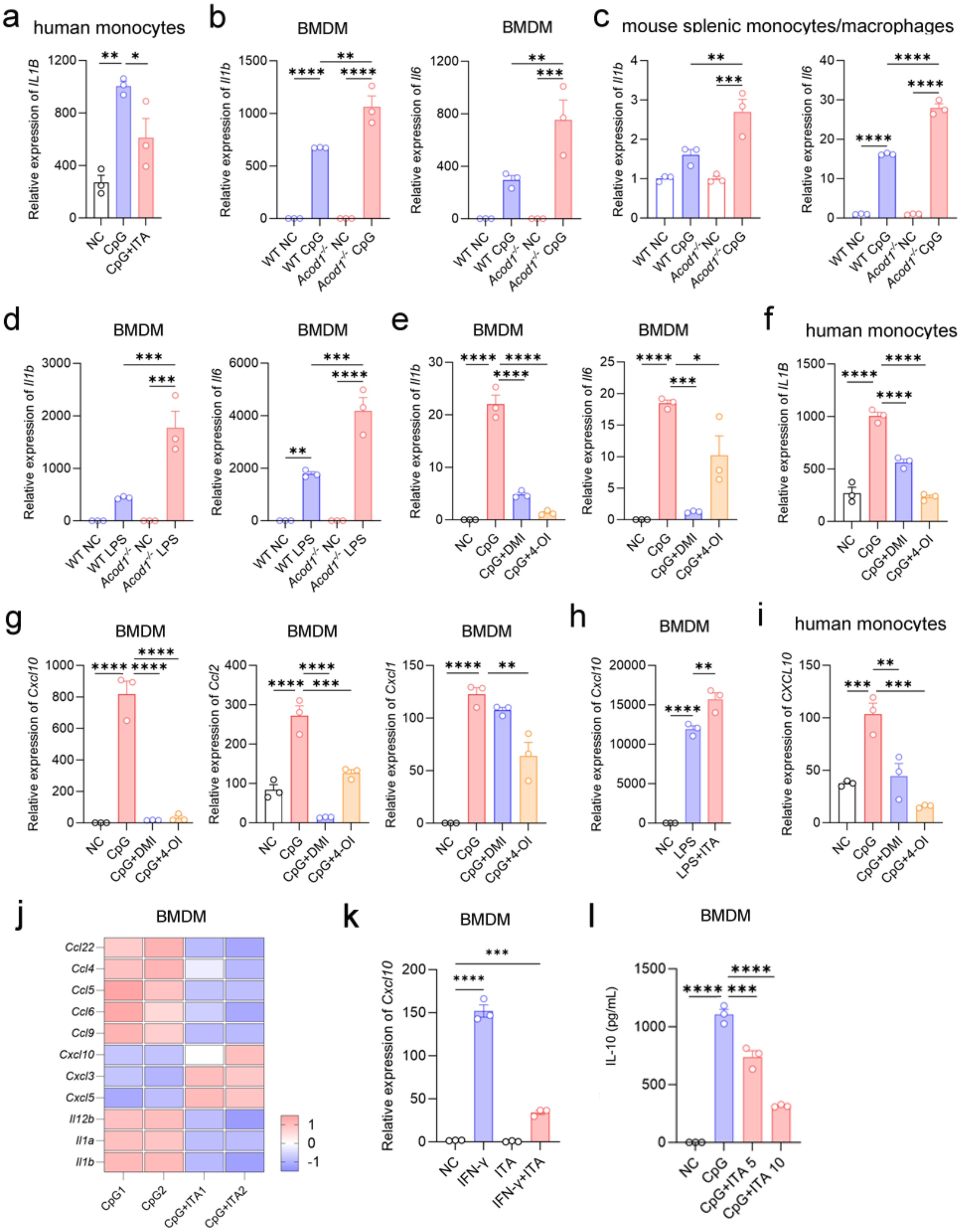

Supplementary Fig. 4 Itaconate differentially regulates cytokines and chemokines in monocytes/macrophages. (a) qPCR analysis of *IL1B* mRNA in human peripheral monocytes pre-treated with 10 mM itaconate for 2 h, followed by 1 μM CpG stimulation for 3 h. (b, c) *Il1b* and *Il6* mRNA in CpG-treated WT or *Acod1*$^{-/-}$ BMDMs (b) or murine spleen monocytes/macrophages (c) (1 μM CpG, 3 h stimulation, n = 3). (d) *Il1b* and *Il6* mRNA in LPS (100 ng/mL)-treated WT or *Acod1*$^{-/-}$ BMDMs (3 h stimulation, n = 3). (e) *Il1b* and *Il6* mRNA in CpG-treated (1 μM, 3 h) BMDMs with 2 h pretreatment of 100 μM DMI/4-OI (n = 3). (f) *Il1B* mRNA in CpG-treated (1 μM, 3 h) human peripheral monocytes with 2 h pretreatment of 100 μM DMI/4-OI (n = 3). (g) *Cxcl10*, *Ccl2*, and *Cxcl1* mRNA in CpG-

treated (1 μM, 3 h) BMDMs with 2 h pretreatment of 100 μM DMI/4-OI (n = 3). (h) *Cxcl10* mRNA in LPS-stimulated (100 ng/mL, 3 h) BMDMs with itaconate pre-treatment (10 mM, 2 h) (n = 3). (i) *CXCL10* mRNA in in CpG-treated (1 μM, 3 h) human peripheral monocytes with 2 h pretreatment of 100 μM DMI/4-OI (3 h). (j) RNA-sequencing analysis of cytokine and chemokine expression in BMDMs pre-treated with 10 mM itaconate for 2 h, followed by 1 μM CpG stimulation for 3 h (n = 2). (k) *Cxcl10* mRNA in IFN-γ-stimulated (10 ng/mL, 3 h) BMDMs with itaconate pre-treatment (10 mM, 2 h) (n = 3). (l) IL-10 levels in supernatants of BMDM pre-treated with itaconate (10 mM, 2 h), followed by treatment of CpG (1 μM, 6 h) (n = 3). Figures are representatives of three independent experiments. The bar plots were presented by mean ± s.e.m. * $p < 0.05$; ** $p < 0.01$; *** $p < 0.001$; **** $p < 0.0001$. 4-OI, 4-octyl itaconate; DMI, dimethyl itaconate.

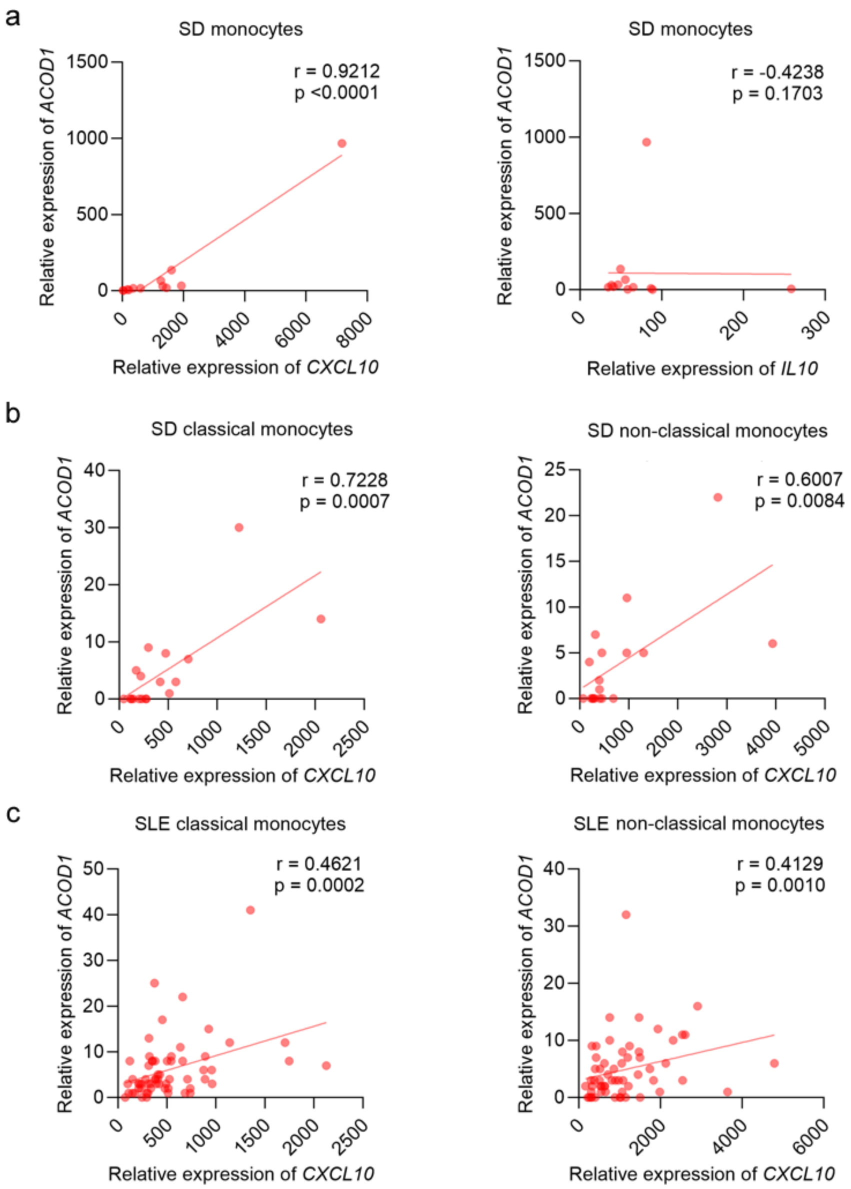

Supplementary Fig. 5 Correlation between *ACOD1* and *CXCL10* expression in monocytes of SD patients. (a) Spearman correlation between *ACOD1* and *CXCL10* or *IL10* expression in monocytes of SD patients (n = 12; analysis using a recently published bulk RNA-seq dataset GSE247993). (b) Spearman correlation in classical and non-classical monocytes of SD patients (n = 18; analysis using a recently published bulk RNA-seq dataset E-GEAD-397). (c) Spearman correlation in classical and non-classical monocytes of SLE patients (n = 61; using E-GEAD-397 dataset).

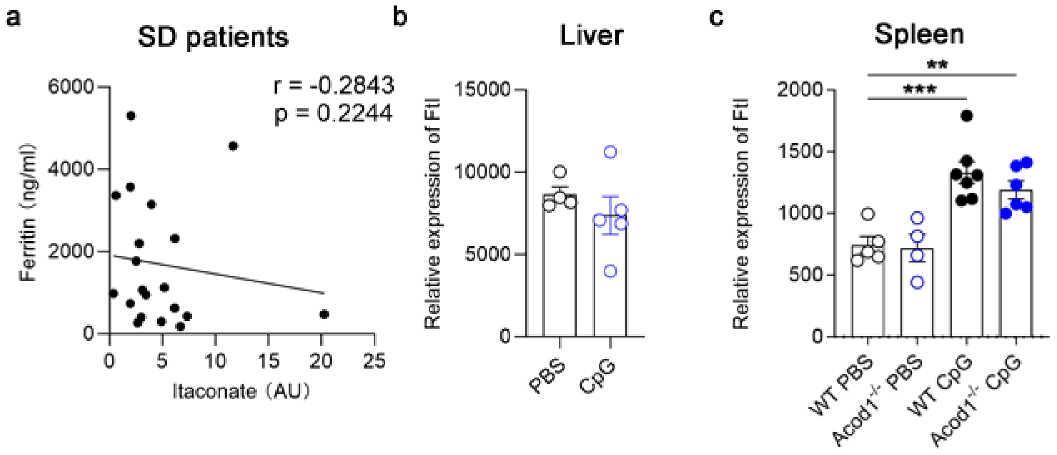

Supplementary Fig. 6. Ferritin levels in SD patients and mouse model of MAS. (a) The correlation analysis between serum levels of ferritin and itaconate (n = 20). (b) The expression of ferritin light chain (*Ftl*) in livers from PBS- (n = 4) or CpG-treated mice (n = 5). (c) The expression of *Ftl* in spleens from PBS- (n = 4-5) or CpG-treated (n = 6-7) WT/*Acod1*$^{-/-}$ mice. The data are presented as mean ± s.e.m. ** $p < 0.01$; *** $p < 0.001$. AU represents arbitrary unit for signal intensity.
